# Is There a Noninvasive Source of MSCs Isolated with GMP Methods with Better Osteogenic Potential?

**DOI:** 10.1155/2019/7951696

**Published:** 2019-11-06

**Authors:** Carla C. G. Pinheiro, Alessander Leyendecker Junior, Daniela Y. S. Tanikawa, José Ricardo Muniz Ferreira, Reza Jarrahy, Daniela F. Bueno

**Affiliations:** ^1^Hospital Sírio-Libanês-Instituto de Ensino e Pesquisa, São Paulo, SP 01308-050, Brazil; ^2^Instituto Militar de Engenharia (IME), Departamento de Ciências de Materiais, Programa de Pós Graduação em Ciências de Materiais, Rio de Janeiro, RJ 22290-270, Brazil; ^3^David Geffen School of Medicine, Division of Plastic and Reconstructive Surgery, University of California Los Angeles (UCLA), Los Angeles, CA, USA

## Abstract

**Background:**

A new trend in the treatment for alveolar clefts in patients with cleft lip and palate involves the use of bone tissue engineering strategies to reduce or eliminate the morbidity associated with autologous bone grafting. The use of mesenchymal stem cells—autologous cells obtained from tissues such as bone marrow and fat—combined with various biomaterials has been proposed as a viable option for use in cleft patients. However, invasive procedures are necessary to obtain the mesenchymal stem cells from these two sources. To eliminate donor site morbidity, noninvasive stem cell sources such as the umbilical cord, orbicularis oris muscle, and deciduous dental pulp have been studied for use in alveolar cleft bone tissue engineering. In this study, we evaluate the osteogenic potential of these various stem cell types.

**Methods:**

Ten cellular strains obtained from each different source (umbilical cord, orbicularis oris muscle, or deciduous dental pulp) were induced to osteogenic differentiation *in vitro*, and the bone matrix deposition of each primary culture was quantified. To evaluate whether greater osteogenic potential of the established mesenchymal stem cell strains was associated with an increase in the expression profile of neural crest genes, real-time qPCR was performed on the following genes: SRY-box 9, SRY-box 10, nerve growth factor receptor, transcription factor AP-2 alpha, and paired box 3.

**Results:**

The mesenchymal stem cells obtained from deciduous dental pulp and orbicularis oris muscle demonstrated increased osteogenic potential with significantly more extracellular bone matrix deposition when compared to primary cultures obtained from the umbilical cord after twenty-one days in culture (*p* = 0.007 and *p* = 0.005, respectively). The paired box 3 gene was more highly expressed in the MSCs obtained from deciduous dental pulp and orbicularis oris muscle than in those obtained from the umbilical cord.

**Conclusion:**

These results suggest that deciduous dental pulp and orbicularis oris muscle stem cells demonstrate superior osteogenic differentiation potential relative to umbilical cord-derived stem cells and that this increased potential is related to their neural crest origins. Based on these observations, and the distinct translational advantage of incorporating stem cells from noninvasive tissue sources into tissue engineering protocols, greater study of these specific cell lines in the setting of alveolar cleft repair is indicated.

## 1. Background

Tissue bioengineering is characterized by the integration of engineering strategies and biological principles with the aim of restoring, maintaining, or improving the function of tissues affected by various pathologies [1, 2]. The main objective of tissue bioengineering is to overcome the limitations of conventional treatments that are based on traditional reconstructive surgery or organ transplantation through the combination of cells with great growth potential (e.g., stem cells), biocompatible delivery vehicles, and growth factors. The goal of many tissue engineering protocols is to create organ and tissue substitutes that exhibit immunologic tolerance and that minimize the disadvantages associated with more traditional techniques [3].

The application of bioengineering principles has rapidly increased in all medical and dental specialties [1, 4]. Congenital malformations associated with cleft and craniofacial syndromes have been extensively studied as part of this expansive research focus. Specifically, tissue engineering approaches to the rehabilitation of the cleft alveolus in patients who are born with complete cleft lip and palate (CLP) have been an area of intense investigation. Currently, the “gold standard” in the treatment of patients with alveolar clefts is the placement of an autologous bone graft. In this surgical procedure, the bone is harvested from the patient—typically from the iliac crest—and used to fill the alveolar cleft [5, 6]. This method, however, has significant drawbacks. For example, the amount of available bone graft donor sites, and the amount of bone that can be procured from these sites, is finite. In cases of large or bilateral clefts, a donor area such as the iliac crest may not provide enough graft material to fill the alveolar cleft. Furthermore, bone resorption in the grafted area may occur, requiring additional procedures. Donor site infection is a reality [7], and, of course, the significant amount of pain that patients experience in the hip region cannot be understated.

Fortunately, with the application of tissue bioengineering principles to this clinical problem, and with our ability to procure autologous stem cells in noninvasive ways, we are now poised to use these cells in innovative ways that might obviate the need for traditional bone grafting and its associated drawbacks. Within this context, mesenchymal stem cells (MSCs) represent a promising biological substrate [1].

MSCs are defined as cells that have the capacity to proliferate and self-renew. They have the ability to respond to external stimuli and give rise to numerous distinct specialized cell lines. MSCs are found in different tissues, are arranged in niches throughout the body, and are responsible for tissue maintenance and repair. MSCs are commonly considered to be of mesodermal origin. Some authors associate various MSC strains with the expression of genes related to embryonic stem cells as well as genes related to the neural crest cell origin [8].

Protocols describing the expansion of MSC populations from umbilical cord isolates, also known as umbilical cord MSCs (UC-MSCs), have been well described. Several authors describe the isolation of UC-MSCs from different components of the umbilical cord, including the cord epithelium and Wharton's jelly. Different types of enzymatic digestion can be used to isolate UC-MSCs, which are characterized by UC-CD73+, CD90+, and CD105+ expression profiles. Some have described that various UC-MSC strains also express embryonic stem cell markers, such as Podocalyxin (Tra-1-60/Tra-1-81), Stage Specific Embryonic Antigen-1 (SSEA-1), and Stage Specific Embryonic Antigen-4 (SSEA-4) [9–11].

Pluripotency markers, such as octamer-binding transcription factor 4 (Oct-4), SRY-box2 (SOX2), and Nanog markers, are not found in UC-MSCs. UC-MSCs are described as adult MSCs, with intermediate characteristics between embryonic stem cells and adult multipotent cells and may have better potential than MSCs isolated from bone marrow (BM) or fat for tissue engineering [12].

Due to their origin, MSCs from exfoliated deciduous teeth, or dental pulp stem cells (DPSCs), are noteworthy: they have a faster proliferation rate than the pulp of permanent teeth and they express primitive cell neuronal and glial markers on their surfaces [8, 13]. The expression of neuronal and glial markers in stem cells from human exfoliated deciduous teeth (SHED) is related to their origin, as they originate from the migration of neural crest cells. These cells play a fundamental role in embryonic development, giving rise to all the tissues of the face, except the enamel of the teeth [8, 13, 14].

Orbicularis oris muscle-derived stem cells (OOMDSCs) have been found to represent a noninvasive source of MSCs for CLP patients, with the potential to reconstruct critical size bone defects in animal models [15]. Facial development, including development of the oral cavity and dental structures, is characterized by epithelial-cell interactions between the craniofacial mesoderm and the neural crest-derived mesenchyme. Therefore, all facial tissues, including DPSCs and OOMDSCs, retain some genes that are expressed in neural crest cells in their cellular population [16].

In this study, we compare the osteogenic potential of three different noninvasive sources of MSCs-DPSCs, OOMDSCs, and UC-MSCs--for use in bone tissue engineering. Specifically, we assess their applicability in a bioengineered alternative to traditional alveolar bone graft surgery in CLP patients. We also quantify the expression profile of neural crest genes SRY-box 9 (SOX9), SRY-box 10 (SOX10), nerve growth factor receptor (NGFR), transcription factor AP-2 alpha (TFAP2a), and paired box 3 (PAX3) in DPSCs, OOMDSCs, and UC-MSCs to determine whether the level of expression of these genes in these MSC populations correlates with their osteogenic potential.

## 2. Materials and Methods

### 2.1. Obtaining, Isolating, and Characterizing Primary Cultures of MSCs

Thirty samples of different tissues were obtained from thirty pediatric patients at our affiliate institutions (deciduous dental pulp, 10 samples; orbicular oris muscle, 10 samples; and umbilical cord, 10 samples). Our research protocol was approved by the Ethics Committee of Hospital Sírio-Libanês, and informed consent was obtained from the legal guardians of all pediatric subjects enrolled in this study. (Of note, these tissues would all be discarded under normal circumstances. The use of these tissues therefore posed no additional burden to the donors.) Cells were isolated according to previously established protocols [15, 17, 18]; however, we added the good manufacturing practice (GMP) grade to the protocols.

For the collection of tissues from a surgical center (muscle fragments and umbilical cords) or dental office (dental pulp), basic care using sterile materials was implemented to avoid contamination. Time from tissue collection to cellular isolation never exceeded 24 hours after collection to avoid cell loss and possible cross-contamination. Our laboratory has standard biosafety certifications used for the handling and processing of human tissues (e.g., anteroom for paramentation and HEPA air filters). From cell isolation to cryopreservation, reagents that were sterile, apyrogenic, and with batch traceability were used. Aerobic, anaerobic, and fungus contamination tests (BactAlert, bioMérieux) and mycoplasma tests (MycoAlert kit, Lonza) were carried out during cell expansion. Any samples with positive results were discarded.

### 2.2. Establishment of Primary Cultures at GMP Laboratory

Our laboratory facilities are regulated by Brazilian laws and resolutions (National Sanitary Surveillance Agency—ANVISA—RDC No 214, February 8, 2018) that regulate advanced cell therapies. According to the local regulatory committee, our laboratory facilities have regular inspections, conduct staff trainings, perform routine equipment maintenance and risk and adverse event assessments, and use fully traceable reagents and processes [19, 20]. We have recommended infrastructure for clean rooms including airflow and air particulate control (HEPA filter) and antechambers for individual protection paramentation. Only human cells can be processed at our advanced cell therapy laboratory site. Moreover, all reagents from cell isolation to cryopreservation are certified, prion-free, and apyrogenic.

#### 2.2.1. DPSCs

The deciduous dental pulp specimens were collected by surgical extraction at a dental office in the Hospital Municipal Infantil Menino Jesus from CLP patients and immediately added to a sterile container with 2 ml of Dulbecco's modified Eagle's medium/Nutrient Mixture F-12 (DMEM-F12; Gibco Invitrogen, Grand Island, NY) solution supplemented with 100 IU/ml penicillin and streptomycin (Penicillin-Streptomycin; Gibco Invitrogen, Grand Island, NY). Afterwards, they were transported to the laboratory of Hospital Sírio-Libanês at 4 to 8°C in a transport box. Deciduous dental pulp was processed on average at 15 hours after initial collection.

In the laboratory, the deciduous dental pulp specimens were washed twice with phosphate-buffered saline (PBS, pH 7.4; Gibco Invitrogen, Grand Island, NY) and digested with a solution containing 1 mg/ml of TrypLE™ Express Enzyme (Gibco Invitrogen, Grand Island, NY) in PBS for 30 minutes at 37°C. After tissue digestion, the samples were centrifuged at 300 × *g* for 5 minutes, and then the pulp was cut into two or more 1 mm^3^ fragments. After these procedures, the cells were cultured in a 12-well plate with each fragment in a separate well.

#### 2.2.2. OOMDSCs

Fragments of the orbicular oris muscle were collected by surgical extraction at Hospital Municipal Infantil Menino Jesus from CLP patients during cheiloplasty and immediately added to a sterile collector tube with 2 ml of DMEM-F12 solution supplemented with 100 IU/ml penicillin and streptomycin. The orbicular oris muscle fragments were processed on average up to 16 hours after collection.

In the laboratory, fragments of the orbicular oris muscle were washed twice with PBS and digested with a solution containing 1 mg/ml of TrypLE™ Express in PBS for 40 minutes at 37°C. After the enzymatic digestion, the samples were centrifuged at 300 × *g* for 10 minutes. The muscle fragments were divided into three parts and cultured in a 12-well plate with each fragment in a separate well. MSCs were expelled from the fragment 10 to 20 days after this procedure.

#### 2.2.3. UC-MSCs

The umbilical cord fragments were collected at Maternidade Amparo Maternal, from mothers who previously elected to donate umbilical cord blood to a cord blood bank. After collecting the blood, the umbilical cord was decontaminated with chlorhexidine (0.12%), and the fragment (5 to 8 cm) was immediately added to a sterile collection tube with 2 ml of PBS solution supplemented with 100 IU/ml of penicillin and streptomycin. The fragments were processed on average up to 16 hours after collection.

In the laboratory, the umbilical cord fragments were washed twice with PBS. For better manipulation of the fragments, the tissue was cut into smaller pieces of two to three centimeters, and then the arteries and veins were removed. The stromal tissue was cut into smaller pieces and added to a Falcon-type tube with a 2.0 mg/ml solution of collagenase NB6 (GMP-SERVA Electrophoresis; Nordmark GmBH, Crescent Chemical) diluted in PBS with 2 mM calcium chloride for two hours at 37°C with continuous movement. To remove the digestion solution, the samples were centrifuged at 300 × *g* for 10 minutes. The digested tissue was resuspended in 10 ml of a DMEM-F12 culture medium supplemented with 15% Characterized Fetal Bovine Serum (FBS; US Origin HyClone™, GE Healthcare Life Sciences, South Logan, UT), 100 IU/ml of penicillin and streptomycin and nonessential amino acid (MEM Non-Essential Amino Acids Solution; Gibco Invitrogen, Grand Island, NY) and cultured in 25 cm^2^ culture flasks. The successful isolation of MSCs was observed between 15 and 20 days after this process. This culture medium was used for cell expansion in all strains (DPSC, OOMDSCs, and UC-MSC) until the cells reached approximately 80-90% confluence.

### 2.3. Cryopreservation

All 30 strains were cryopreserved before the assays using DMEM-F12 diluted 1 : 1 with FBS and 10% dimethyl sulfoxide (DMSO; CryoPur™ 100% DMSO; OriGen). The temperature was gradually decreased by 1°C per minute to -80°C, and the cells were stored at -196°C.

### 2.4. Characterization by Flow Cytometry

For all 30 strains between the 4th and the 5th passage, immunophenotyping was performed by flow cytometry in a FACSCalibur flow cytometer (BD, Becton Dickinson Franklin Lakes, NJ) and analyzed in the CellQuest program (BD, Becton Dickinson Franklin Lakes, NJ). Immunophenotyping allows the characterization of cells at different stages of development through the use of fluorescent monoclonal antibodies against surface markers (antigens).

Cells obtained from cell cultures at a concentration of 1 × 10^6^ cells/100 *μ*l were labeled with the following monoclonal antibodies: CD29-PE, CD31-FITC, CD34-FITC, CD45-PE, CD73-FITC, CD90-FITC, CD105-PE, CD166-PE, IgG-FITC, and IgG-PE isotypes (BD Biosciences, Becton Dickinson, Franklin Lakes, NJ) for 15 minutes at room temperature in the dark. Five hundred microliters of PBS was then added with 3% FBS and incubated for 15 minutes at room temperature in the dark. First, unstained cells were analyzed, and from that analysis, specific isotypes for each antibody were used for staining, with monoclonal antibodies as a negative control for the reaction, and were measured the minimum 5 × 10^5^ events.

### 2.5. Characterization by Cell Differentiation Ability

#### 2.5.1. Osteogenic Differentiation

The 30 strains between the 4th and the 5th passage were induced for osteogenic differentiation. After the culture in the osteogenic medium for 21 days, we assessed *in vitro* formation of bone matrix by assessing areas of culture that were positive for calcium hydroxyapatite.

In a 12-well plate (Corning® Costar®), the cells obtained from each of the 30 strains were seeded at the same density in triplicate (5 × 10^3^ cells). After 24 hours of culture in DMEM-F12, the culture medium was changed to a specific osteogenic induction medium supplemented with growth factors (StemPro® Osteogenesis Differentiation Kit; Gibco Invitrogen, Grand Island, NY).

After twenty-one days in culture, we stained each culture dish with alizarin red S. The wells were washed twice with PBS and fixed with 70% ethanol (Sigma Aldrich, St. Louis, MO) for 30 minutes. After fixation, the wells were stained with 0.2% alizarin red S solution (pH 4.2; Sigma Aldrich, St. Louis, MO) for 30 minutes. For the final wash, each well was washed with PBS (Gibco Invitrogen, Grand Island, NY) three times. We analyzed the formation of mineralized bone extracellular matrix by microscopy (Olympus CKX31).

#### 2.5.2. Adipogenic Differentiation

Primary MSC cultures were cultured in an adipogenic induction medium for eighteen days. After this period, we observed the morphological changes and the formation of intracellular lipid vesicles in the cultured cells.

In a 12-well plate, the cells were seeded at the same density in triplicate (5 × 10^3^ cells). After 24 hours of culture in basal culture medium, the culture medium was changed to the specific adipogenic culture medium supplemented with growth factors (StemPro® Adipogenic Differentiation Kit; Gibco Invitrogen, Grand Island, NY).

For evaluation, the adipogenic induction medium was removed from the cell cultures, and the cells were stained with oil red (Oil Red O, Sigma Aldrich, St. Louis, MO). For the staining, the wells were washed twice with PBS (Gibco Invitrogen, Grand Island, NY) and fixed with 60% isopropanol (Sigma Aldrich, St. Louis, MO) for five minutes at room temperature. After fixation, the cells were stained with oil red (0.5 mg/ml) for 15 minutes under light at room temperature. For the final wash, 60% isopropanol was used once and distilled water twice.

After the staining of the lipid vesicles, the observation of cellular structures was carried out under inverted microscopy (Olympus CKX31).

#### 2.5.3. Chondrogenic Differentiation

To perform chondrogenic differentiation, MSCs were induced to differentiate into chondrocytes after twenty-one days of culture in a chondrogenic induction medium supplemented with growth factors.

In a 12-well plate, the cells were seeded at the same concentration in triplicate (5 × 10^4^ cells). After 24 hours of culture in a basal culture medium, the culture medium was changed to the specific chondrogenic differentiation medium supplemented with growth factors (StemPro® Chondrogenic Differentiation Kit; Gibco Invitrogen, Grand Island, NY).

For the evaluation of chondrogenic differentiation, we performed staining with alcian blue after 21 days in differentiation conditions to identify the proteoglycan (extracellular matrix) released by the chondrocytes.

The induction medium was removed from the cell cultures, which were washed twice with PBS (Gibco Invitrogen, Grand Island, NY) and fixed with 4% formaldehyde (Sigma Aldrich, St. Louis, MO) for 20 minutes at room temperature. After fixation, the cells were stained with 1 mg/ml of alcian blue (Sigma Aldrich, St. Louis, MO) for two hours in the dark at room temperature. For the final wash, hydrochloric acid (0.1 M) was used once and with PBS twice.

### 2.6. Quantification of Mineralized Bone Matrix

As one of the objectives of this research was to evaluate the potential for osteogenic differentiation in MSCs from three different sources, 10 primary cultures of DPSCs, 10 primary cultures of OOMDSCs, and 10 primary cultures of UC-MSCs were induced to osteogenic differentiation in a 24-well plate. For this assay, 2.5 × 10^3^ cells were seeded in triplicate between the 4th and the 5th passage.

For the initial seeding, the basal culture medium was used after 24 hours when the cells were already adhered to the bottom of the culture plate. The osteogenic induction was initiated by changing the basal culture medium with osteogenic induction medium (StemPro® Osteogenic Differentiation Kit; Gibco Invitrogen, Grand Island, NY). The osteogenic differentiation process was analyzed after 0, 3, 7, 14, and 21 days of culture in osteogenic differentiation medium.

For analysis of bone extracellular matrix formation, the culture medium was removed and 0.5 mg/ml of alizarin red S (pH 4.2; Sigma Aldrich, St. Louis, MO) diluted in PBS (Gibco Invitrogen, Grand Island, NY) was added to each well. Subsequently, the cells were incubated under light for 30 minutes at room temperature.

After 30 minutes, 200 *μ*l of 20% methanol solution (Sigma Aldrich, St. Louis, MO) and 10% acetic acid diluted in PBS (Sigma Aldrich, St. Louis, MO) were added to each well and then incubated for 15 minutes in the dark to solubilize the crystal formed by alizarin red S staining. The plate was then agitated for approximately five minutes for complete solubilization. The solution was then transferred to a 96-well plate for the measurement of osteogenic differentiation in a plate reader (Infinite 200 PRO; Tecan, Switzerland). The results were analyzed according to a calibration curve previously performed for each cell type.

### 2.7. mRNA Extraction

RNA extraction was performed when the primary samples presented MSC characteristics such as being undifferentiated, being in the same stage and passages that were used in the osteogenic differentiation experiments. The MSCs were induced to differentiate into osteoblasts in vitro and used in the experiments as described previously.

Total RNA was extracted from cells cultured in vitro using the RNAeasy Mini Kit (QIAGEN, Hilden, Germany). This kit was developed for the extraction of total RNA from small amounts of starting material. It is a gold standard method that combines the selective binding properties of a silica gel membrane with microcentrifuge velocity.

The protocol used for the extraction technique was provided by the manufacturer.

A Bioanalyzer Kit (Agilent RNA 6000 Nano Kit) was used to evaluate RNA quality. This process allowed us to verify the integrity (RIN) and precise quantification of the samples before any application dependent on the amount of RNA was obtained.

cDNA synthesis was conducted with the SuperScript™ VILO™ cDNA Synthesis Kit (Invitrogen) following the protocol provided by the manufacturer.

### 2.8. Quantitative Real-Time PCR Analysis

By quantitative real-time PCR (qRT-PCR), we evaluated the expression levels of genes related to the expression of neural crest cell markers SOX9, SOX10, NGFR, TFAP2a, and PAX3. The analysis of the gene expression used in our study represents the relative quantification of the genes of interest using an endogenous control (normalizing gene). In this study, the genes SDHA and HPRT1 were used as endogenous controls (supplementary [Supplementary-material supplementary-material-1]).

For qRT-PCR, we used SYBR Green PCR Master Mix (Applied Biosystems, Warrington, UK) for the amplification and quantification of nucleic acids. Reactions were performed in triplicate with a final volume of 20 *μ*l for each reaction. We used 10 *μ*l of the real-time SYBR Green PCR Master Mix (2x), 2 *μ*l of the cDNA sample at a concentration of 0.2 *μ*g/*μ*l, 2 *μ*l of a first sense primer, 2 *μ*l of a reverse primer, and 4 *μ*l of ultrapure water. The quantification was performed by using the Applied Biosystems 7300 Real-Time PCR System, according to the following steps: 95°C for two minutes, 40 cycles at 95°C for 15 seconds and 60°C for 30 seconds, and a subsequent dissociation step.

### 2.9. Statistical Analysis

To analyze the osteogenic differentiation between DPSC, OOMDSCs, and UC-MSC, we used a two-way ANOVA statistic test with repeated measures for a single factor (time). When multiple comparisons of means were necessary, the Bonferroni post hoc test was used. The expression of the genes to be used as normalization factors was determined, that is, genes commonly expressed in MSCs. Expression analysis was calculated from the efficiency of each probe, elevated to the Ct delta of the reference minus the Ct delta of the sample of each gene, as shown in the constitutive (Δctref) − (Δct sample) formula proposed by Pfaffl in 2001 [21].

To evaluate whether there was a difference between the groups regarding gene expression, the Kruskal-Wallis test was used. When multiple comparisons were required, the Dunn test was used. A type I (*α*) probability of error of 0.05 was considered in all inferential analyses. Descriptive and inferential statistical analyses were performed with SPSS software version 21 (SPSS 21.0 for Windows) with a significance level of *α* = 0.05.

## 3. Results

We collected 10 deciduous dental pulp samples, 12 orbicularis oris muscle samples, and 25 umbilical cord samples, with MSC isolation rates of 100%, 83.3%, and 40%, respectively. The failure to achieve a 100% isolation rate was due to microbiological contamination for the orbicularis oris muscle and was due to technical problems in the establishment of the protocols described in the literature for the umbilical cord samples. We used a collagenase developed for use in GMP and we need to increase its concentration over the previously described protocols that used non-GMP collagenase [18, 22–24].

Osteogenic differentiation was performed in each of the 10 different MSC strains, but a cell pool was not performed with respect to the individuality of each MSC strain during the osteogenic differentiation process.

**Figure 1:**
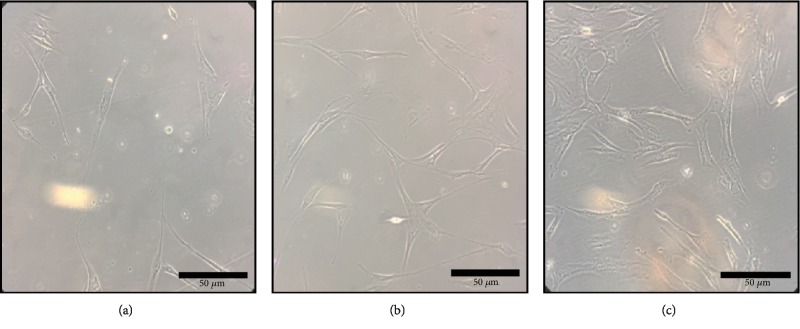
Morphology of adherent cells after isolation from corresponding tissue (sources): (a) orbicular oris muscle-derived stem cell (OOMDSC); (b) dental pulp stem cell (DPSC); (c) umbilical cord mesenchymal stem cell (UC-MSC). Similarity in the fibroblastoid morphology among the three different strains is observed.

Samples collected from deciduous dental pulp and orbicular oris muscle underwent enzymatic processing and expelled MSCs 15 days after cell culture (Figure 1). UC-MSCs were obtained after the validation of the MSC isolation protocol and were observed in culture between 20 and 25 days after the enzymatic digestion procedure (Figure 1).

### 3.1. Characterization of MSC Strains

All DPSC, OOMDSC, and UC-MSC primary cultures showed a very similar surface marker expression profile by flow cytometry analysis, as shown in Table 1. The expression of the markers CD29, CD31, CD34, CD45, CD73, CD90, CD105, and CD166 is plotted in the supplementary material.

**Figure 2:**
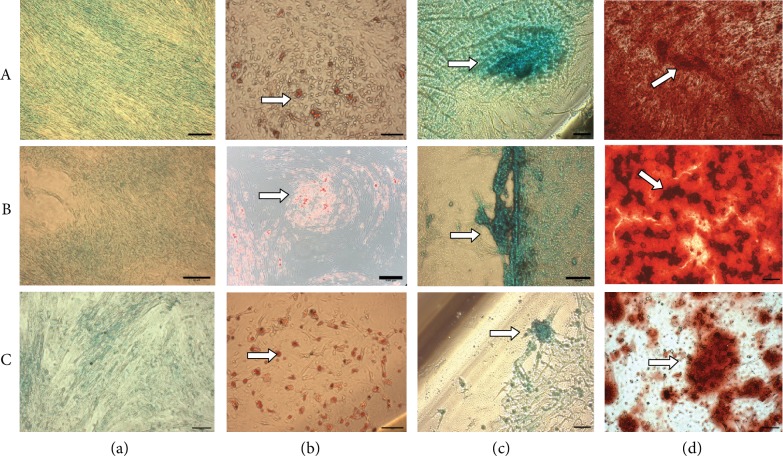
Multilineage differentiation in vitro. Row A: OOMDSC; row B: DPSC; and row C: UC-MSC. (a) The control group of undifferentiated strains. (b) Adipogenic differentiation after eighteen days of induction and staining with oil red; white arrows show the fat vesicles. (c) Chondrogenic differentiation after 3 weeks of induction, stained with alcian blue; white arrows show the extracellular matrix formation—mucopolysaccharides. (d) Osteogenic differentiation after 3 weeks of OOMDSC induction, stained with alizarin red S; white arrows show the extracellular matrix deposition.

All primary cultures of DPSCs (*n* = 10), OOMDSCs (*n* = 10), and UC-MSCs (*n* = 10) were able to differentiate into osteoblasts, chondrocytes, and adipocytes (see Figure 2).

### 3.2. Quantification of Bone Extracellular Matrix

**Figure 3:**
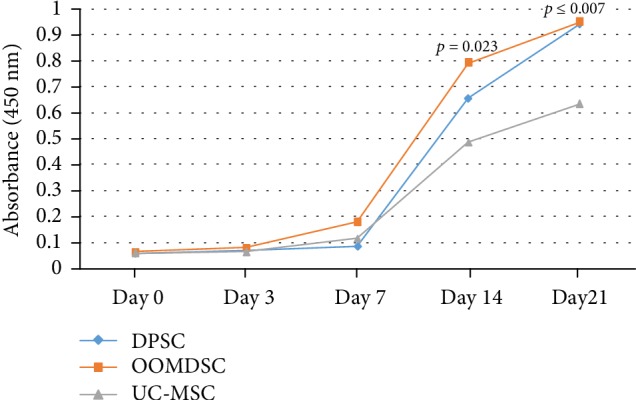
Quantitative measurement of the extracellular bone matrix stained with alizarin red S. Graphical representation of the measurement of the extracellular bone matrix deposited during osteogenic differentiation induction at 0, 3, 7, 14, and 21 days, showing the beginning of the deposition of extracellular matrix after 7 days of induction in vitro with increases on days 14 and 21.

We observed osteogenic differentiation after 21 days in all 30 strains obtained; however, we observed a higher deposition of extracellular matrix in OOMDSCs and DPSCs, with a statistically significant difference compared to UC-MSCs (Figure 3). No statistically significant difference was observed when comparing DPSCs to OOMDSCs.

When we observed the initial phase of osteogenic differentiation on days 3 and 7, there was no difference in the production of extracellular matrix between the groups. On day 14, there was deposition of extracellular matrix in the OOMDSC strains compared with the UC-MSC strains that was statistically significant (*p* = 0.023). However, on day 21 of osteogenic differentiation, when all undifferentiated cells were already in the osteoblast stage producing extracellular matrix, higher extracellular matrix deposition was observed in the OOMDSC and DPSC groups than in the UC-MSC group (*p* = 0.005 and *p* = 0.007, respectively) (Figure 3). There was no difference between OOMDSCs and DPSCs in the formation of extracellular matrix on the 21st day of cell differentiation.

### 3.3. Gene Expression Evaluation for Neural Crest Cell Markers

**Figure 4:**
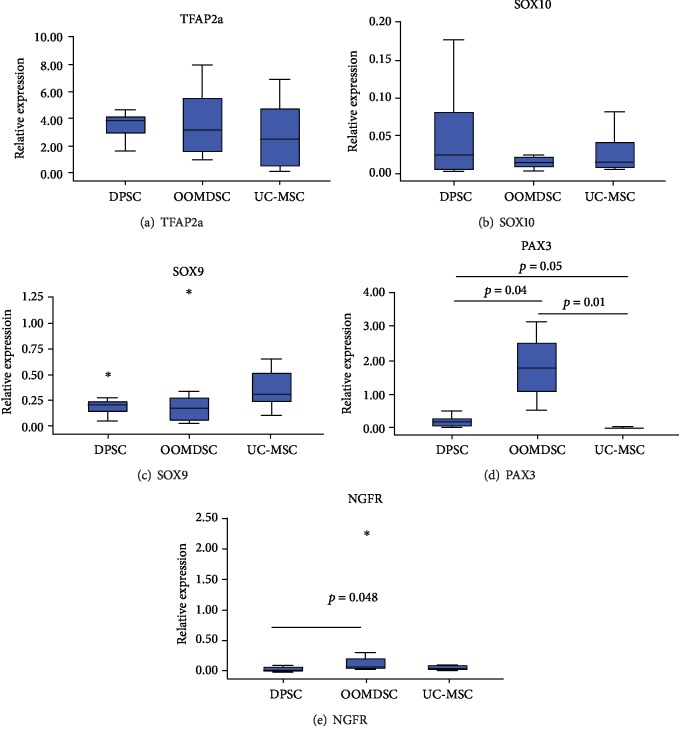
Neural crest expression in MSCs: relative expression of 5 neural crest genes in undifferentiated DPSC, OOMDSC, and UC-MSC strains. This experiment was repeated with three replicates for each sample (*n* = 10). The data are presented as the mean +/− (∗ represents the outlier data).

We observed greater expression of PAX3 in the OOMDSC strains than in the DPSC (*p* = 0.04) and UC-MSC (*p* < 0.001) strains. There was also a trend of increased PAX expression in DPSCs when compared to UC-MSCs (*p* = 0.05) (Figure 4(d)).

The NGFR gene was expressed in all strains obtained from DPSCs, OOMDSCs, and UC-MSCs (Figure 4(e)). Significantly greater expression of this gene was observed in OOMDSCs than in DPSCs (*p* = 0.048) and in UC-MSCs than in DPSCs (*p* = 0.046). No statistically significant difference was observed when comparing the expression profile of the NGFR gene between OOMDSCs and UC-MSCs (*p* > 0.999).

The expression of the TFAP2a, SOX10, and SOX9 genes in all MSC strains was also demonstrated, but there was no significant difference in their expression when the three distinct strains were compared to one another (*p* = 0.654, *p* = 0.761, and *p* = 0.124, respectively) (Figures 4(a)–4(c)).

## 4. Discussion

The search for new sources of MSCs as an alternative to the isolation of MSCs from BM has been increasing in the last decade, mainly as a strategy for developing regenerative medicine solutions to clinical problems. Since 2000, studies have described the isolation of stem cells from different sources, such as dental pulp, muscle, fat, and umbilical cord [13, 15, 17, 18, 25–27].

The objective of this study was to determine if there was a correlation between various sources of MSCs and their osteogenic potential. Specifically, we compared the osteogenic potential of cells of neural crest origin to MSCs isolated from the umbilical cord. Our focus on neural crest origin is particularly relevant since the neural crest is responsible for the development of bone and craniofacial connective tissue, and one of the leading potential clinical applications of this tissue engineering paradigm is in the treatment of patients with CLP-associated bone defects.

Considering the different potential sources of stem cells, umbilical cord and deciduous dental pulp are noteworthy because they are easy to obtain and are considered a noninvasive source. Umbilical cord tissue is discarded at birth, and during early infancy, primary teeth undergo a process of natural exfoliation for the exchange of deciduous for permanent dentition. Both sources of stem cells have great potential for the formation of other tissues, such as bone, muscle, fat, and cartilage [17, 18, 28–35]. Therefore, the cells isolated from these tissues can be cryopreserved in biological storage banks for future use. In the case of patients with CLP, these cells may be thawed for potential use to heal alveolar clefts via a bone tissue engineering approach [36, 37].

For patients with craniofacial malformations, especially CLP patients, another noninvasive source of MSCs is the orbicularis oris muscle. Small fragments of this muscle can be obtained during cheiloplasty surgery and, in fact, are often discarded during the cleft lip repair. MSCs which are capable of osteogenic differentiation can be isolated from these tissues [15].

The results of this study demonstrate that, using good manufacturing practice (GMP) protocols, it is possible to isolate MSCs from deciduous tooth pulp, orbicularis oris muscle, and umbilical cord stroma to be used in clinical interventions, corroborating other studies in the literature. Our results provide further support for the practice of cryopreserving these MSCs for later use in clinical trials and approved cell therapies [38–40]. Our laboratory facilities adhere to all Brazilian laws and regulations governing the use of human tissues in research and advanced cell therapies [19, 20, 37]. Moreover, our group has previously tested genetic stability of the cells used in this study through passage 18; no chromosomal abnormalities at the 1st or at the 18th passage were observed [15, 41]. In obtaining MSCs from deciduous dental pulp, we did not encounter any issues with our GMP laboratory protocols; however, during the establishment of OOMDSC and UC-MSC strains, we had some problems with microbiological contamination and validating the protocols previously described in the literature. To decrease the microbiological contamination in the OOMDSC and UC-MSC strains, antibiotics were added to the culture medium at the time the source tissues were obtained and placed in culture. Additionally, the time between tissue collection and GMP processing to ultimately obtain the MSCs was reduced (from an average of 24 hours to an average of 16 hours). These strategies helped us obtain MSC strains free of contamination with characteristic fibroblastic morphology and adherence to plastic, as recommended by the International Society for Cellular Therapy (ISCT) [42].

In our study, we used bovine serum in cell culture due to our observation of a decrease in cell proliferation when a xeno-free culture medium was used (unpublished data). Alternatives such as platelet lysate or human serum might be more applicable to translational studies. Further investigation of the effects of alternative human-derived culture products on osteogenic differentiation and gene expression is warranted.

Initially, we had some issues in the isolation of MSCs derived from the umbilical cord stroma since there are several isolation protocols available in the literature [11, 18, 22, 43]. These various protocols call for different methods to dissect and remove the arteries and veins, either by digesting only the Wharton jelly or by simply explanting a fragment of the cord for processing. The protocol that is implemented can affect the number of cells obtained and their potential for differentiation into bone, cartilage, or fat and may alter their expression of some cell surface markers [11, 44]. Among the compartments of the cord fragment used to isolate UC-MSCs, Wharton's jelly stands out as the best option, and in this present study, Wharton's jelly digested with 2 mg/ml collagenase with 2 mM calcium chloride was used to obtain the UC-MSCs under GMP conditions. We have demonstrated the capacity of UC-MSCs to differentiate in the three mesodermal lines, which is consistent with previous reports [11, 18, 22–24].

The immunophenotypic expression profile of MSCs was determined by the analysis of a set of surface antigen markers in these cells. Research on MSCs derived from dental pulp describes the use of different flow cytometer panels to characterize these cells [45, 46]. Some studies showed the expression of surface antigens for anti-CD117, anti-STRO-1, anti-CD105, anti-CD73, and anti-CD90 antibodies but did not show the expression of the CD45, CD34, and CD14 hematopoietic markers and the CD31 endothelial markers [8, 47]; however, there is no consensus in the literature on which markers should be used [8, 47, 48].

After freezing and thawing, the immunophenotypic characterization of DPSCs, OOMDSCs, and UC-MSCs revealed the presence of cells expressing high levels of MSC markers such as CD105 (endoglin), CD73 (ecto-5′-nucleotidase), CD44 (HCAM), CD90 (Thy-1), CD166 (ALCAM), and CD29 and lacking the expression of CD31 (PECAM-1), CD34, and CD45. The expression of CD117 (c-kit) was low, as has also been reported in other studies with cultures of different MSCs [13, 15]. CD117 is a primitive marker and can be expressed in the first passages of SHED cultures. In this work, we performed cell characterization by flow cytometry analysis at the 4th cell passage, which may be the reason for the low expression of this marker [8, 49].

One of the important biological properties in the characterization of MSCs is their ability to differentiate into at least 3 tissue types of the mesenchymal lineage. Thus, in the present study, primary cultures of all the studied groups demonstrated the capacity for osteogenic, adipogenic, and chondrogenic differentiation when exposed to the appropriate differentiation medium. All experiments used frozen and thawed cells with preserved ability to differentiate into the three different cell lines. This demonstrates that MSCs obtained from different tissues can be cryopreserved after isolation and stored until later use. Our experiments used cells up to 5 passages and stored for up to 2 years in liquid nitrogen at -196°C. All our DPSC, OOMDSC, and UC-MSC strains meet the criteria for MSCs according to the requirements of the ISCT [42].

Since the osteogenic potential of MSCs is affected by different factors, such as tissue origin (source) and heterogeneity of the cell population [14], the preselection of subpopulations of cells with greater osteogenic potential is a promising strategy for the complete translation of MSC-based therapies into clinical practice. In this study, we observed the osteogenic potential of DPSCs, OOMDSCs, and UC-MSCs and observed that DPSCs and OOMDSCs had better osteogenic potential than UC-MSCs. Furthermore, we observed that it is possible to isolate these MSCs under GMP conditions and that the cryopreserving and thawing of these cells had no deleterious effect on their osteogenic differentiation.

Fanganiello and colleagues investigated the expression of molecular markers that might be predictive of the osteogenic potential of MSCs, comparing populations of two different sources of MSCs (lipoaspirate and dental pulp). Their results demonstrated that SHED had an intrinsically greater osteogenic potential compared to adipose tissue-derived mesenchymal stem cells (AD-MSCs) when both cell lines were exposed to the same controlled *in vitro* induction system. The transcriptome analysis of these cells during osteogenic differentiation revealed that the upregulated IGF2 gene expression profile may be one of the best predictors of gene expression before and during the onset of osteogenic differentiation in MSCs *in vitro* [50]. In our study, we demonstrated that DPSCs have greater osteogenic potential than UC-MSCs.

Another interesting finding in our study was the similar behavior between strains obtained from DPSCs and OOMDSCs. When these strains were induced to osteogenic differentiation, a significant difference was not observed. One hypothesis for this finding would be that both tissues (sources) have the same origin from neural crest cells [16, 27]. To test this hypothesis, we analyzed the expression of genes directly linked to the neural crest cell population in the three groups of primary cells proposed in this study. TFAP2a, SOX9, and SOX10 genes were expressed by DPSCs, OOMDSCs, and UC-MSCs, but these genes were not differentially expressed between these strains to any significant degree [51–53].

In one published comparison between the osteogenic potential of DPSCs, UC-MSCs, AD-MSCs, MSCs isolated from peripheral blood, and MSCs isolated from the periodontal ligament (PDLSC), DPSCs demonstrated a greater capacity for osteogenic differentiation [54]. Our results also showed a better differentiation capacity in DPSCs than in UC-MSCs. However, in contrast to our findings, in which a significant difference in SOX9 expression was not observed in our tested cell lines, in Trivanović's study, patient DPSCs expressed higher levels of SOX9 than the other MSC lines, even when all of them differentiated into the chondrogenic lineage, where SOX9 staining is presented as a specific marker [54]. However, in cells from the umbilical cord, there was a tendency (without statistical difference) to have greater expression of SOX9, which may be a better alternative when using for chondrogenic differentiation, optimizing protocols for specific use in therapies such as repair of cartilage.

In our results, the expression profile of the PAX3 gene was higher in the primary cultures of DPSCs and OOMDSCs than in the UC-MSCs, corroborating the literature and demonstrating that these lineages maintain a greater expression of PAX3 in their cell population. This observation suggest that these cell lines retain features of neural crest cells that are predisposed to a greater osteogenic differentiation potential [55, 56]. During the development of each line derived from the neural crest, several regulatory genes are involved, including PAX. The genes of the PAX family are essential transcription factors that play important roles during organogenesis and participate in important stages of this process, such as cell migration, cell proliferation, and cell differentiation [57]. PAX3 gene expression is present in immature neural crest cells and in neural cells [56]. In our study, only the PAX3 gene had a different gene expression profile among the cells obtained from the different sources, demonstrating higher expression in OOMDSCs and DPSCs than in UC-MSCs. Since neural crest cells are the origin of all facial tissues except for tooth enamel [55, 58], we suggest that PAX3 is the best marker of neural crest cells for testing samples of MSCs to identify the cell population with a greater predisposition for osteogenic differentiation. In the literature, the PAX3 gene is expressed in myogenic precursor cells at an embryonic stage of development [59]. Because we used orbicularis oris muscle fragments of the lip as a source of MSCs, isolated during cheiloplasty surgery performed in infants approximately 3-6 months of age, there was a greater possibility of finding more premature MSCs with a high expression of PAX3. In the pulp of deciduous teeth, as described in the literature, there is a heterogeneous MSC niche that expresses premature markers [8, 14]. Corroborating this fact, our results demonstrated a high expression of the PAX3 gene in DPSCs.

In the literature, NGFR gene expression in MSCs is unclear but has been shown to be involved in the survival and differentiation of neuronal cells *in vitro* and plays an important role in neuronal development [60]. These genes can be expressed in bone marrow (BM) cells but not in hematopoietic or endothelial cells [61]. The NGFR antigen has also been described on the earlier BM stromal component in the development of the embryo before the onset of BM activity and in 7 to 11% of the cells of the adherent layer of bone marrow mesenchymal stem cell (BM-MSC) cultures in the long term, suggesting that NGFR antibodies may also be present in primitive MSCs. In 2010, Quirici et al. described the presence of this marker in mesenchymal cells derived from adipose tissue, observing that the cell population that retains this marker has greater clonogenic potency and ability to differentiate into bone [62]. Our results demonstrated the expression of this gene in DPSC, OOMDSC, and UC-MSC lines. However, in our results, we observed that the expression of NGFR in OOMDSCs and UC-MSCs was higher than that of DPSCs. In some studies, CD271 (NGFR) has been described as a selective marker for the purification and characterization of MSCs isolated from BM [63, 64]; however, there is no description in the literature of the expression of this marker in DPSCs. As a consequence, Mikami and colleagues attempted to define the expression of CD271 in DPSCs to elucidate its role in MSCs. They demonstrated that DPSCs have a CD271+/CD90+/CD44+/CD45− expression profile in 2.4% of the cell population. Consistent with our results, this marker was weakly expressed in the DPSC population. The multilineage differentiation potential (osteogenic, adipogenic, chondrogenic, and myogenic) of CD271+/DPSC was compared to that of the CD271−/DPSC population. The results demonstrated the inhibition of the osteogenic capacity of the CD271+/SHED population when compared to the CD271−/DPSC culture, demonstrating lower levels of calcium and alkaline phosphatase. Therefore, DPSCs expressing lower levels of NGFR (CD271−) have superior osteogenic differentiation potential, and over time, the expression of NGFR decreases in CD271+/DPSCs (Mikami et al. [65]). Our findings corroborate the results obtained by Mikami et al. in 2011, suggesting that DPSCs express low levels of the NGFR gene and have a great osteogenic differentiation potential. On the other hand, OOMDSCs were also shown to have a high potential for osteogenic differentiation, and, conversely, it was the cell line that expressed high levels of the NGFR gene [65]. Studies in the literature define the marker CD271 (NGFR) as a potentially specific cell surface marker for a precursor subpopulation of MSCs [63]. However, no study has thus far demonstrated the actual correlation of this marker with proliferative cell potential and *in vitro* and *in vivo* differentiation capabilities. The fact that OOMDSCs exhibit a higher expression profile of the NGFR gene and demonstrate the same osteogenic potential of DPSCs suggests a specific role for this gene in the process of osteogenic differentiation. Furthermore, there are reports in the literature that demonstrate that the greatest potential for osteogenic differentiation of MSCs from different sources is associated with the expression of neural crest genes in the undifferentiated mesenchymal cell populations [51, 65].

According to our findings and relevant published data, we suggest that the NGFR marker may not be a good predictive marker for selecting the best source of MSCs for use in bone tissue engineering. However, we propose that the PAX3 gene may be a potential marker that predicts the osteogenic potential of MSCs obtained from different sources. In our study, higher expression levels of this gene were observed in MSCs that demonstrated greater osteogenic potential. We therefore conclude that DPSCs and OOMDSCs, in part, as indicated by their PAX3 expression, are the best sources of MSCs to be used in bone tissue engineering for CLP patients. These cells can be isolated and cryopreserved under GMP conditions for use in regenerative medicine and therefore represent a very viable substrate for use in translational studies.

## 5. Conclusions

The best sources to obtain MSCs for bone tissue engineering for CLP patients are dental pulp and orbicular oris muscles. MSCs obtained from these tissues have better osteogenic potential than those obtained from umbilical cord. High expression of the PAX3 gene can be a good marker in predicting which tissues would provide the most ideal MSC strains for use in bone tissue engineering. Our results suggest that the superior osteogenic potential observed in DPSCs and OOMDSCs is due to their neural crest cell origins. Based on these observations, further study of the clinical applicability of MSCs isolated from noninvasive sources, such as DPSCs and OOMDSCs, in innovative translational bone tissue engineering protocols to repair alveolar bone grafts in CLP patients is called for.

## Figures and Tables

**Table 1:** Characterization of the profile of DPSCs, OOMDSCs, and UC-MSCs.

Cellular type	In vitro analysis		Immunophenotype			
	Multipotency		Marker		Positive population (%)	Standard deviation (+/−)
DPSC	Osteogenic	+	CD29	+	90	4.9
	Chondrogenic	+	CD31	−	0.4	0.2
	Adipogenic	+	CD34	−	0.2	0.1
			CD45	−	0.5	0.2
			CD73	+	90.1	0.9
			CD90	+	97	0.7
			CD105	+	94	2
			CD166	+	91.6	1.3

OOMDSC	Osteogenic	+	CD29	+	96.3	1.3
	Chondrogenic	+	CD31	−	0.3	0.1
	Adipogenic	+	CD34	−	1.2	0.4
			CD45	−	0.2	0.1
			CD73	+	94	0.6
			CD90	+	97.8	1.3
			CD105	+	92.6	2.1
			CD166	+	90	5

UC-MSC	Osteogenic	+	CD29	+	90.2	4
	Chondrogenic	+	CD31	−	0.1	0.1
	Adipogenic	+	CD34	−	0.1	0.1
			CD45	−	0.1	0.1
			CD73	+	94.1	4.8
			CD90	+	97.7	2
			CD105	+	90	1.0
			CD166	+	91.6	1.1

## Data Availability

The datasets used during the current study are available from the corresponding author upon reasonable request.

## Supplementary Materials

Supplementary Materials Supplementary material 1: all gene sequences used in the neural crest gene profile. Supplementary material 2: the R1, R2, and R3 regions were a population of mesenchymal stem cells: R1: OOMDSC, R2: UC-MSC, and R3: DPSC. The histograms represent the profile markers in the population selected: black line: OOMDSC; green line: UC-MSC; and pink line: DPSC. All strains presented the same profile marker: positive reaction to CD29, CD73, CD90, CD105, and CD166 and negative reaction to CD31, CD34, and CD4.
