# Supplementary material for: Is There a Noninvasive Source of MSCs Isolated with GMP Methods with Better Osteogenic Potential?
Source: Stem Cells Int. 2019 Nov 6;2019:7951696. doi: 10.1155/2019/7951696 (PMC6875366; doi:10.1155/2019/7951696)
Supplement: Supplementary Materials — Supplementary material 1: all gene sequences used in the neural crest gene profile. Supplementary material 2: the R1, R2, and R3 regions were a population of mesenchymal stem cells: R1: OOMDSC, R2: UC-MSC, and R3: DPSC. The histograms represent the profile markers in the population selected: black line: OOMDSC; green line: UC-MSC; and pink line: DPSC. All strains presented the same profile marker: positive reaction to CD29, CD73, CD90, CD105, and CD166 and negative reaction to CD31, CD34, and CD4. [file 7951696.f1.docx]

Supplementary Materials

Supplementary material 1: All gene sequences used in the neural crest gene profile.

| **Gene** | **Primers** |  |
| --- | --- | --- |
| **SOX 10** | R – CATATAGGAGAAGGCCGAGTAGA | NM_006941.3 |
|  | F – CCTCACAGATCGCCTACACC |  |
| **SOX 9** | R – CTGTAGGCGATCTGTTGGGG | NM_000346.3 |
|  | F – AGCGAACGCACATCAAGAC |  |
| **TFAP2A** | R - GACCCGGAACTGAACAGAAGA | NM_001032280.2 |
|  | F – CTCCGCCATCCCTATTAACAAG |  |
| **PAX3** | R – CTCGGATTTCCCAGCTGAAC | NM_181461.3 |
|  | F – AAGCCCAAGCAGGTGACAAC |  |
| **NGFR** | R – TGTCGCTGTGGAGTTTTTCT | NM_002507.3 |
|  | F – ACATAGCCTTCAAGAGGTGGA |  |
| **SDHA*** | R – CCACCACTGCATCAAATTCATG | NM_004168.2 |
|  | F - TGGGAACAAGAGGGCATCTG |  |
| **HPRT1*** | R – GGCCTCCCATCTCCTTCATC | NM_000194.2 |
|  | F – CATTATGCTGAGGATTTGGAAAG |  |

Supplementary material 2: The dot plots and histograms by flow cytometry analyses.


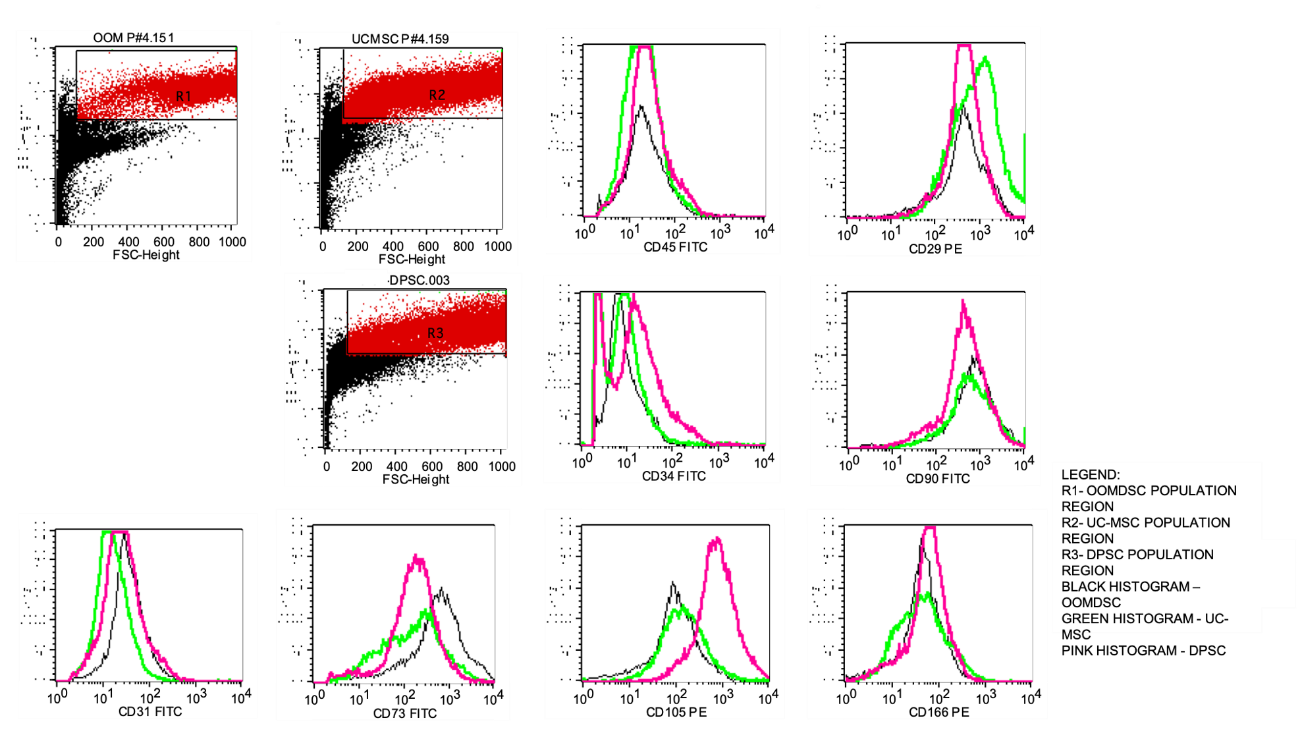


Supplementary material 2: The R1, R2 and R3 regions were a population of mesenchymal stem cells, R1: OOMDSC, R2: UC-MSC and R3: DPSC. The histograms represent the profile markers in the population selected, black line: OOMDSC; green line: UC-MSC and pink line: DPSC. All strains presented the same profile marker, positive reaction to CD29, CD73, CD90, CD105 and CD166, and negative reaction to CD31, CD34 and CD4
